# Epinephrine evokes shortening of human airway smooth muscle cells following β_2_ adrenergic receptor desensitization

**DOI:** 10.1152/ajplung.00444.2021

**Published:** 2022-07-05

**Authors:** Brian T. Deeney, Gaoyuan Cao, Sarah Orfanos, Jordan Lee, Mengyuan Kan, Blanca E. Himes, Vishal Parikh, Cynthia J. Koziol-White, Steven S. An, Reynold A. Panettieri

**Affiliations:** ^1^Rutgers Institute for Translational Medicine and Science, Rutgers, The State University of New Jersey, New Brunswick, New Jersey; ^2^The Joint Graduate Program in Toxicology, Department of Pharmacology and Toxicology, Rutgers-Ernest Mario School of Pharmacy, Rutgers, The State University of New Jersey, Piscataway, New Jersey; ^3^Department of Biostatistics, Epidemiology and Informatics, Perelman School of Medicine, University of Pennsylvania, Philadelphia, Pennsylvania; ^4^Department of Pharmacology, Rutgers-Robert Wood Johnson Medical School, Rutgers, The State University of New Jersey, Piscataway, New Jersey

**Keywords:** adrenergic receptors, airway smooth muscle, asthma, bronchospasm, catecholamines

## Abstract

Epinephrine (EPI), an endogenous catecholamine involved in the body’s fight-or-flight responses to stress, activates α_1_-adrenergic receptors (α_1_ARs) expressed on various organs to evoke a wide range of physiological functions, including vasoconstriction. In the smooth muscle of human bronchi, however, the functional role of EPI on α_1_ARs remains controversial. Classically, evidence suggests that EPI promotes bronchodilation by stimulating β_2_-adrenergic receptors (β_2_ARs). Conventionally, the selective β_2_AR agonism of EPI was thought to be, in part, due to a predominance of β_2_ARs and/or a sparse, or lack of α_1_AR activity in human airway smooth muscle (HASM) cells. Surprisingly, we find that HASM cells express a high abundance of *ADRA1B* (the α_1_AR subtype B) and identify a spontaneous “switch-like” activation of α_1_ARs that evokes intracellular calcium, myosin light chain phosphorylation, and HASM cell shortening. The switch-like responses, and related EPI-induced biochemical and mechanical signals, emerged upon pharmacological inhibition of β_2_ARs and/or under experimental conditions that induce β_2_AR tachyphylaxis. EPI-induced procontractile effects were abrogated by an α_1_AR antagonist, doxazosin mesylate (DM). These data collectively uncover a previously unrecognized feed-forward mechanism driving bronchospasm via two distinct classes of G protein-coupled receptors (GPCRs) and provide a basis for reexamining α_1_AR inhibition for the management of stress/exercise-induced asthma and/or β_2_-agonist insensitivity in patients with difficult-to-control, disease subtypes.

## INTRODUCTION

Asthma defines a syndrome characterized by recurrent airway inflammation and nonspecific airway hyperresponsiveness (AHR) to a wide range of endogenous or exogenous stimuli (1, 2). According to the Centers for Disease Control and Prevention, over 25 million people in the United States had asthma in 2019, with more than half of the individuals exhibiting uncontrolled disease. As a cornerstone therapy, inhaled bronchodilators (short- and long-acting combined) that target β_2_-adrenoceptors (β_2_ARs), which are G protein-coupled receptors (GPCRs) expressed on human airway smooth muscle (HASM) cells, reverse or prevent airflow obstruction—a root cause of asthma morbidity and mortality (3, 4). Of note, for patients with difficult-to-control, severe asthma and/or β_2_-agonist insensitivity, parenteral epinephrine (EPI) is considered as a supplementary add-on therapy, especially to treat anaphylaxis in children (5).

Epinephrine (or adrenaline), a stress hormone released through the hypothalamic-pituitary-adrenal axis, binds both β_2_- and α_1_-adrenoceptors (α_1_ARs) with a high affinity (6, 7). Classically, in the body’s fight-or-flight responses, EPI activates α_1_ARs expressed on various organs to evoke wide-ranging physiological functions (8, 9). The functional role of EPI on α_1_ARs in HASM remains unclear, however, and decreased plasma EPI levels contribute to AHR by decreasing basal β_2_AR stimulation (10, 11). Indeed, reversal of HASM shortening, either with β_2_-agonists or EPI, occurs by binding to the β_2_AR that couples to a stimulatory G protein (G_αs_), activating adenylate cyclase to generate 3′,5′-cyclic adenosine monophosphate (cAMP) (12). Increased cAMP stimulates protein kinase A that then mediates multiple downstream signals to decrease intracellular calcium ([Ca^2+^]_i_) and myosin light chain phosphorylation (pMLC) levels—the latter by the actions of decreased myosin light chain kinase (MLCK) activity and/or increased myosin light chain phosphatase (MLCP) activity-promoting airway smooth muscle relaxation (13–15).

Historically, EPI has served as an add-on bronchodilator for asthma exacerbations (16), but EPI can also activate the α_1_AR to induce airway constriction (17–19). Furthermore, activation of α_1_AR with methoxamine evokes bronchoconstriction in subjects with asthma that is abrogated with prazosin, an α_1_AR antagonist (20, 21). Whether α_1_AR activation plays a role in asthma or whether blocking the α_1_AR has therapeutic value remains unclear (22–24).

Given the limitations of β_2_-agonist therapy in asthma, including drug tolerance or β_2_AR tachyphylaxis with repeated use of β_2_-agonists (25–28), and because EPI can signal through both the β_2_AR and α_1_AR (7, 8), here we considered a plausible therapeutic value of α_1_AR antagonism in preclinical models of β_2_-agonist insensitivity (29–31). Specifically, we posited that EPI induces bronchoconstriction by preferentially activating α_1_ARs expressed on HASM cells, following β_2_AR tachyphylaxis. We demonstrated that, under experimental conditions that induce β_2_AR inhibition or desensitization, EPI evoked [Ca^2+^]_i_, pMLC, and HASM cell shortening. Pretreatment with an α_1_AR antagonist doxazosin mesylate (DM) prevented EPI-induced [Ca^2+^]_i_, pMLC, and HASM cell shortening. These data suggest that EPI-induced HASM cell shortening is α_1_AR-dependent within the context of β_2_AR desensitization. Collectively, our results support that α_1_AR inhibition could serve as a therapeutic target for asthma subtypes, including β_2_-agonist insensitive and stress/exercise-induced obstructive lung disease.

## METHODS

### HASM RNA-Seq Data

We used RNA-seq results from a previously published study that is available in the Gene Expression Omnibus under accession number GSE94335 (32). Briefly, this data set consisted of primary HASM cells derived from age- and sex-matched fatal asthma (*n* = 9) and nonasthma (*n* = 8) lung donors. Normalized read counts for the vehicle control condition based on DESeq2 output of the matrix of raw mapped read counts were obtained for the α_1_AR and β_2_AR genes.

### Materials

Unless otherwise stated, all chemicals and drugs were obtained from Sigma Aldrich (St. Louis, MO). Primary antibodies against pMLC (phosphorylation at Thr^18^/Ser^19^) and GAPDH were purchased from Cell Signaling Technologies (Danvers, MA), MLC from EMD Millipore (Burlington, MA), and α_1_AR from Abcam (Cambridge, UK). For Western blot analyses, IRDye 800CW donkey anti-rabbit and IRDye 680RD donkey anti-mouse were purchased from LI-COR Biosciences (Lincoln, NE). For immunofluorescence analyses, Alexa Fluor 488-conjugated AffiniPure Donkey anti-rabbit IgG [711-545-152] and Rhodamine Red-X conjugated AffiniPure Donkey anti-mouse IgG [715-295-151] were purchased from Jackson ImmunoResearch (West Grove, PA).

### Isolation and Culture of HASM

HASM cells were derived from tracheas obtained from de-identified lung donors procured from the International Institute for the Advancement of Medicine (Edison, NJ) or from the National Disease Research Interchange (Philadelphia, PA); these are not subjected to Rutgers IRB approval. Cell isolation was performed as previously described (33). HASM cells were cultured in Ham’s F-12 medium supplemented with 10% FBS, 100 U/mL penicillin, 0.1 mg/mL streptomycin, and 2.5 mg/mL amphotericin B. For all experiments, HASM cells were serum-deprived for 24 h and used within the first four passages to ensure the proper smooth muscle phenotype (33, 34). Donor demographics for all studies are summarized in Supplemental Table S1.

### Immunofluorescent Staining of Human Bronchus

Immunofluorescent studies were performed on 7-µm thick cryosections of human bronchial tissue. The cold acetone/methanol fixed sections were washed with PBS and treated with a blocking solution (3% BSA and 1:20 diluted FcR Blocking Reagent, MACS Miltenyi Biotec Cat. No: 120-000-442) for 30 min. The sections were then incubated with rabbit anti-α_1_AR (Abcam [ab3462]) and mouse anti-α-smooth muscle actin (Sigma [A2547])) at 4°C overnight (diluted in 1% BSA/PBS) and detected with Alexa Fluor 488-conjugated AffiniPure Donkey anti-rabbit IgG and Rhodamine Red-X conjugated AffiniPure Donkey anti-mouse IgG, respectively. Nonimmune serum served as a negative control, and DAPI was used to stain the nucleus. The stained sections were visualized with a Nikon epifluorescence microscope. All experiments were performed in tissue from a minimum of five distinct donor lungs.

### Immunoblot Analysis

For pharmacological inhibitions of β_2_ARs, HASM cells were treated for 10 min with 10 μM propranolol, before stimulation with increasing concentration of EPI (0.01–10 μM). To induce β_2_AR desensitization, HASM cells were treated for 18 h with 10 μM albuterol (29–31); the cell monolayers were washed to remove albuterol and then stimulated for 10 min with 10 μM EPI or carbachol (CCh). To assess the mechanistic action of EPI on α_1_AR, cells were treated for 10 min with 1 μM doxazosin mesylate (DM), before EPI and CCh stimulation. The cell monolayers were then scraped and collected after adding 0.1% final concentration of perchloric acid. Cells were pelleted, lysed, and incubated overnight at 4°C with NuPage reducing agent and sample buffer. Proteins were separated using SDS-PAGE and transferred to nitrocellulose membranes. Phosphorylation of MLC (pMLC at Thr^18^/Ser^19^) was assessed and normalized to total MLC band densities. Protein bands were detected using near-infrared conjugated secondary antibodies with Li-Cor Odyssey CLX, and the intensity of the protein bands was calculated using Image Studio v. 5.2. A total of five individual nonasthma donor cell lines were used in all experiments.

### Calcium Mobilization Analysis

Cells were grown in 48-well plates and cytosolic calcium levels were measured, as previously described (35). All measurements were performed in serum-free conditions after treating the cell monolayers with or without either propranolol (10 μM, 10 min) or albuterol (10 μM, 18 h). Briefly, cells were incubated with calcium-binding Fluo 8 dye for 1 h (kept in the dark). In some experiments, DM (1 μM, 10 min) or diluent was added 10 min before EPI (25 μM). Real-time fluorescence intensity was then measured in a fluorescent plate reader over 120 s (Clariostar BMG Labtech). Five donor cell lines were run with four technical replicates per experimental condition.

### Magnetic Twisting Cytometry

Magnetic twisting cytometry (MTC) was used to assess the single-cell contractility (i.e., HASM cellular contraction and relaxation), as previously described (34, 36, 37). Briefly, an RGD-coated ferrimagnetic microbead (4.5 μm in diameter) functionalized to the cytoskeleton through cell surface integrin receptors was magnetized and twisted by an external magnetic field that varied sinusoidally in time. Forced bead motions (lateral bead displacements in response to the resulting oscillatory torque) were detected optically with a spatial resolution of ∼5 nm, and their changes were monitored, in real-time, in response to 10 μM EPI. Cell stiffness is computed as the ratio of specific torque to lateral bead displacements and expressed in units of Pascal per nanometer (Pa/nm). For each individual HASM cell, changes in stiffness in response to EPI were normalized to their respective stiffness before EPI addition.

### Bronchodilation Assay

Human precision-cut lung slices (hPCLS) were prepared as previously described (29), and agonist-induced changes in the airway luminal area were measured using Image-Pro Plus software (v. 6.0; Media Cybernetics). For these studies, hPCLS were first contracted with carbachol (10 μM, 10 min), washed, and allowed to return to baseline followed by stimulation with EPI (10 μM). hPCLS were desensitized with Salmeterol (1 μM, 48 h), then stimulated with EPI (10 μM), washed, and then stimulated with carbachol (10 μM). hPCLS were washed and treated with the β_2_AR inhibitor propranolol (10 μM, 30 min), and then stimulated with EPI (10 μM). Bronchodilation and bronchoconstriction were calculated as the percent increase or decrease from the most recent baseline measurement. Refer to Supplemental Fig. S1 for a time course depiction.

### Statistical Analysis

GraphPad Prism software (GraphPad) was used to determine if samples were normally distributed using the Kolmogorov–Smirnov test. Unless otherwise stated, statistical comparisons were done with two-tailed, paired Student’s *t* tests for comparison between two conditions, or an analysis of variance (ANOVA) followed by post hoc *t* tests with Tukey’s correction for multiple comparisons. *P* values of <0.05 were considered statistically significant. Data are represented as means ± SE with a minimum of three biological replicates per condition.

## RESULTS

### HASM Cells Express α_1_-Adrenoceptors

**Figure 1.**
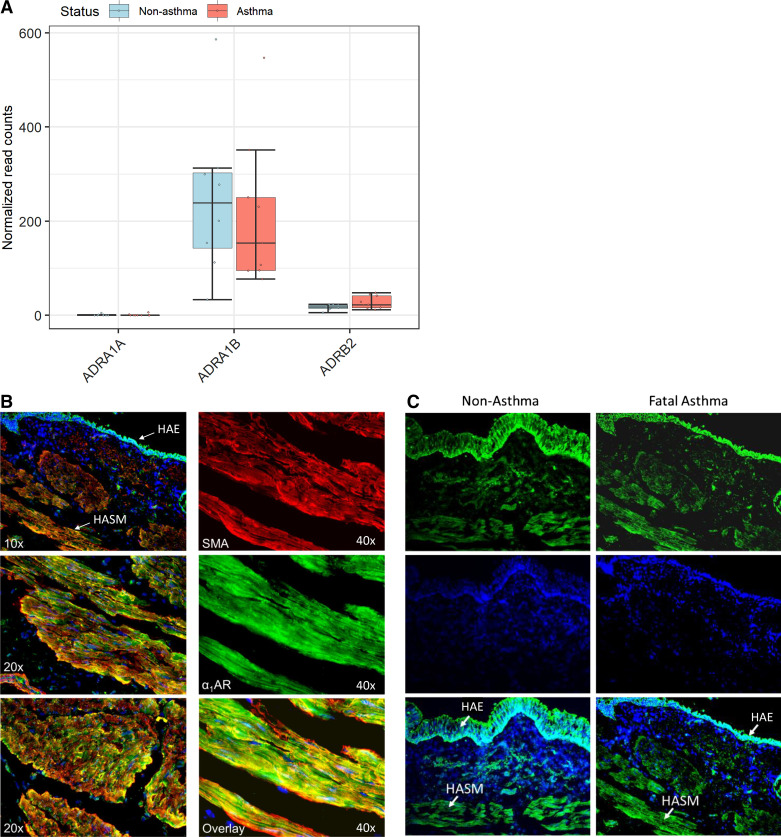
Bronchial tissue expression of α_1_AR on HASM. *A*: *α_1_ar* (*adra1b*) (*n* = 9 asthma and *8* nonasthma donors) mRNA transcripts from RNAseq (represented as normalized read count). *B*: immunofluorescence staining of human bronchus tissue. *C*: comparison of nonasthma and fatal asthma derived donor bronchial tissue. HAE, human airway epithelial cells; HASM, human airway smooth muscle; SMA, antismooth muscle actin = red, anti-α_1_AR = green, DAPI = blue. α_1_AR, α_1_-adrenergic receptor.

According to RNA-Seq results for HASM cells derived from age- and sex-matched fatal asthma or nonasthma lung donors, both the *ADRA1A* and *ADRA1B* genes (encoding α_1_AR subtypes A and B) were expressed. As shown in Fig. 1*A*, *ADRA1B* had mean normalized counts of 212 in asthma-donor-derived HASM cells and 247 in nonasthma-donor-derived HASM cells, values that were approximately 10-fold higher than those of *ADRB2*, which encodes the β_2_AR (mean normalized counts of 28 and 17 in asthma- and nonasthma-donor-derived HASM cells, respectively). Of note, levels for *ADRA1B* and *ADRB2* varied according to donor, but were not significantly different by asthma status. In all samples, expression levels for *ADRA1A* were low (mean normalized counts were ∼1).

To assess the distribution of α_1_ARs in an intact airway, and their localization to HASM cells, bronchial tissue sections were co-stained for α_1_AR and α-smooth muscle actin. As shown in Fig. 1*B*, we detected protein levels of α_1_ARs on epithelial and smooth muscle cells of human airways. Staining of the α_1_AR was comparable in both cell types, with little difference between asthma- and nonasthma-donor-derived airways (Fig. 1*C*). Collectively, these data demonstrate that α_1_ARs are present on HASM cells.

### Epinephrine Evokes Calcium Signal and pMLC upon β_2_AR Blockade in HASM Cells

Stimulation of α_1_ARs can induce airway constriction, but the cellular mechanisms remain unclear. The role for α_1_ARs in asthma pathogenesis is equally unclear. Here we posited that, under a specific condition of β2AR tachyphylaxis in asthma pathobiology, endogenous or exogenous catecholamine(s) can induce bronchoconstriction by activating α_1_ARs expressed on HASM. To test this hypothesis, we used EPI, an agonist of both α_1_- and β_2_-adrenoceptors, in HASM cells treated with and without a pharmacological inhibitor of β_2_AR, propranolol (prop).

**Figure 2.**
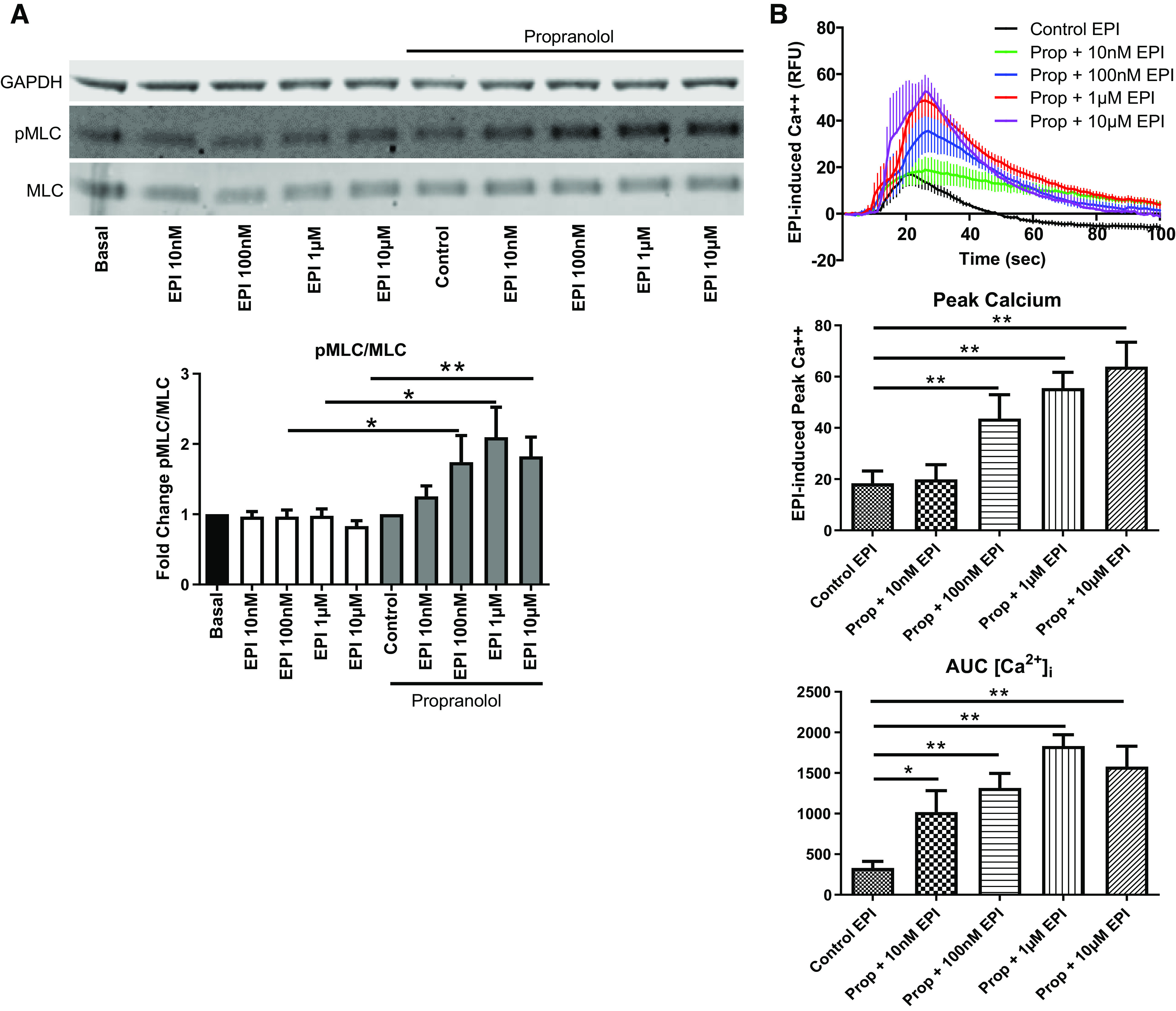
EPI induces MLC phosphorylation and cytosolic calcium flux in HASM after β_2_AR blockade. *A*: EPI (10 nM–10 µM, 10 min) does not increase pMLC in diluent treated HASM; however as EPI concentration increases, pMLC increases in propranolol-pretreated (10 nm–10 μM, 10 min) HASM (values are means ± SE, *n* = 8) (*P ≤ 0.05, **P ≤ 0.01). *B*: EPI-induced cytosolic calcium levels are increased after β_2_AR blockade with propranolol (Prop) in HASM as measured by peak calcium and AUC (values are means ± SE, *n* = 4–9) (*P ≤ 0.05, **P ≤ 0.01). AUC, area under the curve; β_2_AR, β_2_-adrenergic receptor; EPI, epinephrine; HASM, human airway smooth muscle; pMLC, myosin light chain phosphorylation.

As phosphorylation of myosin light chain (pMLC) is a pivotal signaling event mediating agonist-induced HASM shortening, we first measured pMLC in response to increasing doses of EPI. As expected, in HASM cells treated with diluent (controls), EPI had little effect on pMLC levels (Fig. 2*A*); EPI evokes bronchodilation via preferentially activating the β_2_AR expressed on HASM cells (12, 16). In contrast, prop (10 μM, 10 min) appreciably increased pMLC levels in response to EPI; the increases were dose-dependent, and maximum pMLC levels were achieved with 1–10 μM EPI (Fig. 2*A*).

Previous studies showed that α_1_AR couples to G_αq/11_ and evokes intracellular calcium mobilization via phospholipase C (38–40). As such, we measured EPI-induced intracellular calcium levels ([Ca^2+^]_i_) using Fluo 8. As shown in Fig. 2*B*, in HASM cells pretreated with prop, EPI (0.01–10 μM) markedly increased [Ca^2+^]_i_ in a dose-dependent manner. A small, transient increase in [Ca^2+^]_i_ was also detected with 10 μM EPI in cells treated with diluent controls. Compared with control cells, EPI-induced [Ca^2+^]_i_, as shown by peak relative fluorescence units (RFUs) and the integrated area under the curve (AUC), was significantly greater in prop-treated cells, at all tested doses of EPI (Fig. 2*B*). Collectively, these results suggest that EPI can act as a procontractile agonist within the context of β_2_AR inhibition.

### Epinephrine-Induced pMLC Is Mediated via α_1_ARs

**Figure 3.**
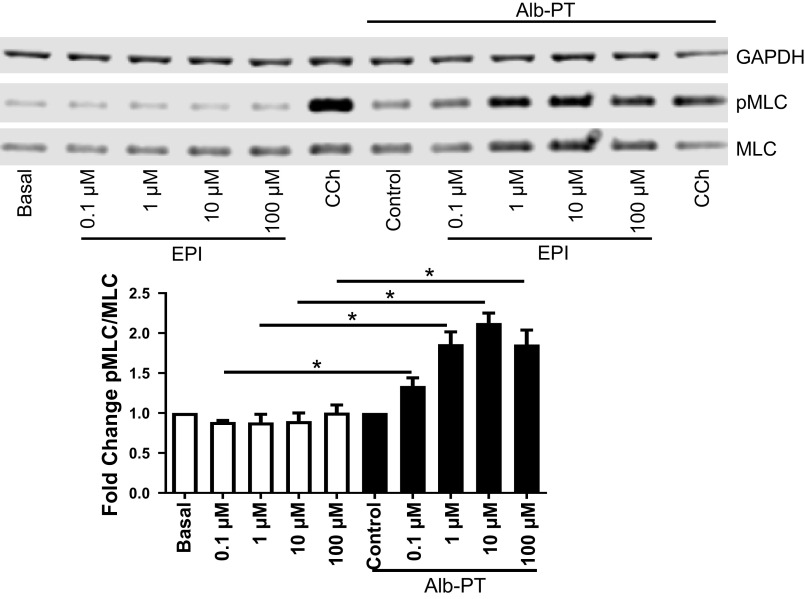
EPI induces MLC phosphorylation in HASM under β_2_AR desensitizing conditions. As EPI (0.1–100 µM, 10 min) concentration increases, pMLC increases in β_2_AR desensitized [Alb-PT (albuterol pretreated) 10 μM, 24 h] HASM but not in nondesensitized (basal). Carbachol (CCh; 20 µM, 10 min) was used as a positive control to elicit pMLC (values are mean ± SEM, *n* = 4 cell lines) (*P ≤ 0.05). β_2_AR, β_2_-adrenergic receptor; EPI, epinephrine; HASM, human airway smooth muscle; pMLC, myosin light chain phosphorylation.

**Figure 4.**
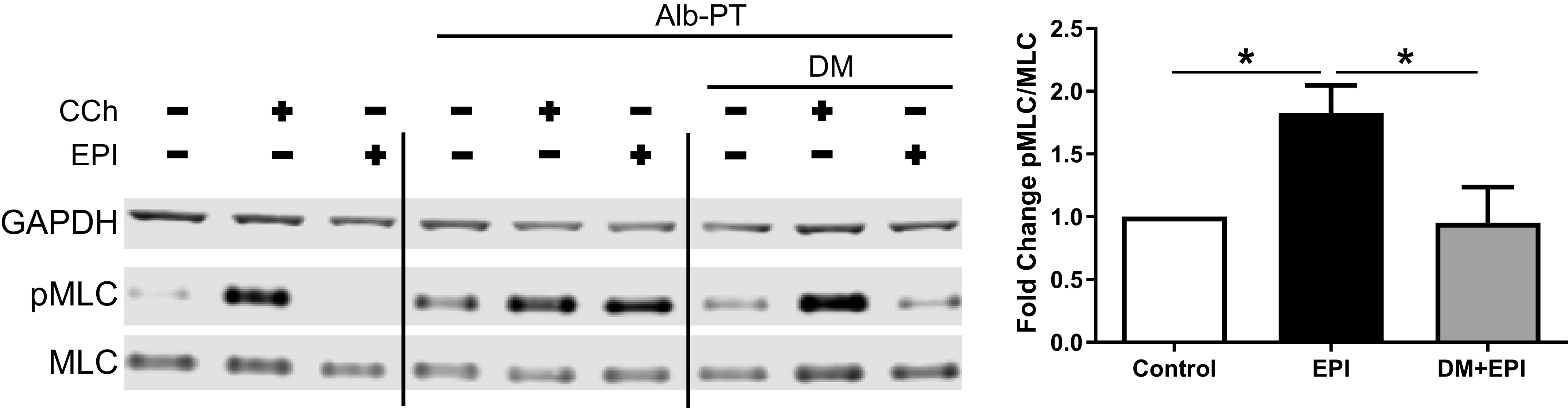
Inhibition of α_1_AR abrogates EPI-induced pMLC in HASM following β_2_AR desensitization. Representative immunoblot of HASM pretreated with DM (1 µM, 10 min pretreatment) before EPI (10 µM, 10 min) stimulation. Band densities measured by fold change pMLC relative fluorescence units normalized to total MLC relative to Alb-PT control (values are means ± SE, *n* = 5–13 experiments from 5 unique cell lines) (*P ≤ 0.05). β_2_AR, β_2_-adrenergic receptor; CCh, carbachol; EPI, epinephrine; HASM, human airway smooth muscle; pMLC, myosin light chain phosphorylation.

To mimic the effect of β_2_AR tachyphylaxis in vitro, HASM cells were treated with 10 μM albuterol for 18 h (29–31). As shown in Fig. 3, HASM cells treated with diluent control showed no appreciable increase in pMLC in response to increasing doses of EPI. In contrast, in HASM cells treated with albuterol (i.e., β_2_-agonist insensitive cellular model), EPI markedly increased pMLC, in a concentration-dependent manner (Fig. 3). Of note, EPI-induced increases in pMLC levels were inhibited by pretreating the β_2_-agonist insensitive cells with DM, an antagonist to α_1_AR. Incubation with DM had little effect on carbachol (CCh)-induced pMLC levels (Fig. 4). These results suggest that EPI-induced HASM contraction is mediated by selective activation of α_1_ARs expressed on HASM cells.

### Inhibition of α_1_ARs Abrogates EPI-Induced HASM Shortening

**Figure 5.**
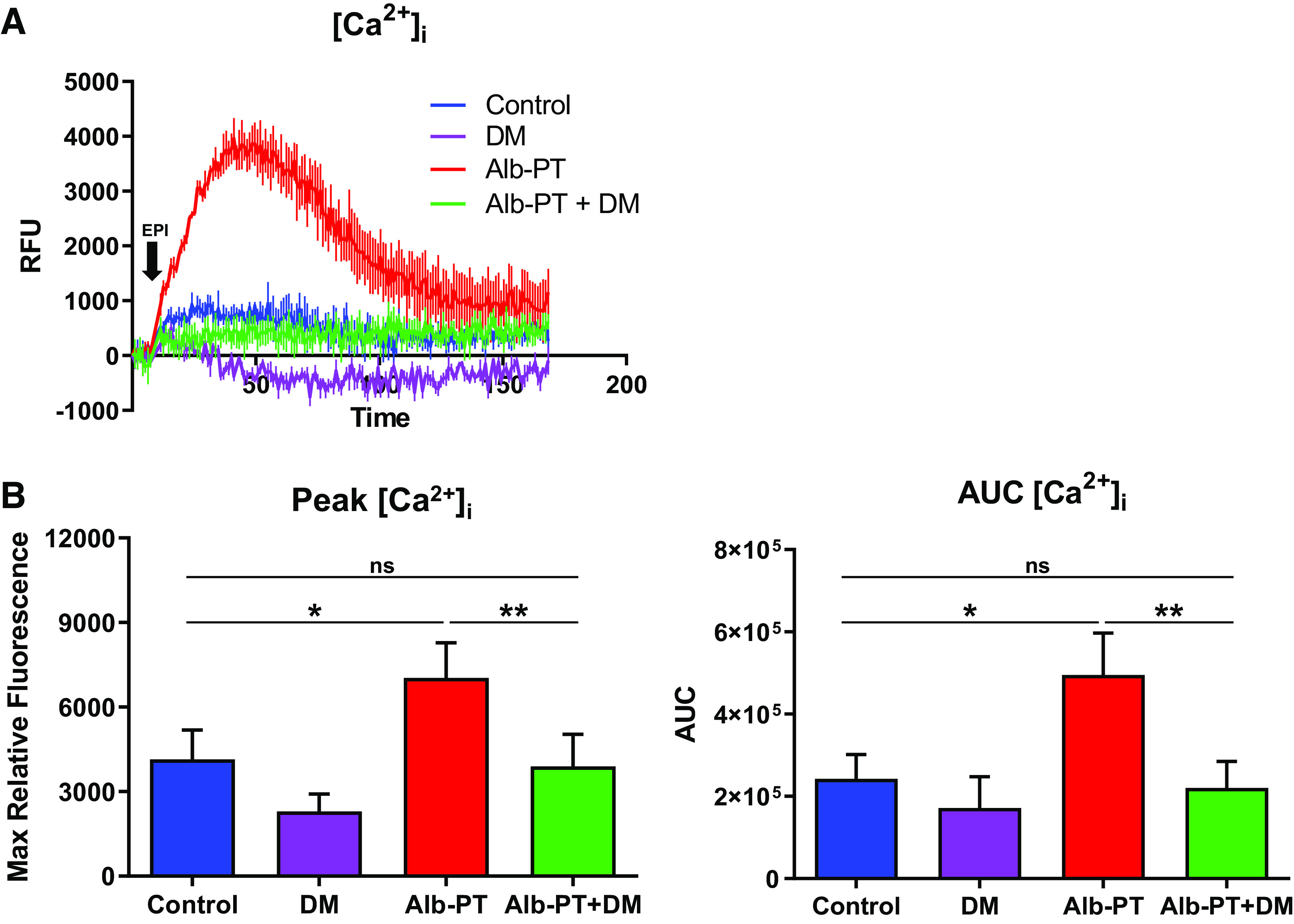
Inhibition of α_1_AR decreases EPI-induced cytosolic calcium levels following β_2_AR desensitization of HASM. *A*: representative graph of EPI-induced cytosolic calcium flux. *B*: inhibition of α_1_AR (DM, 1 µM, 10 min pretreatment) before EPI (25 µM) stimulation decreases cytosolic calcium levels ([Ca^2+^]_i_) as measured by peak relative fluorescence units (peak RFUs) and integrated area under the curve (AUC) after albuterol-induced β_2_AR desensitization (values are means ± SE, *n* = 5 unique cell lines run in triplicate) (*P ≤ 0.05, **P ≤ 0.01). α_1_AR, α_1_-adrenergic receptor; β_2_AR, β_2_-adrenergic receptor; DM, doxazosin mesylate; EPI, epinephrine; HASM, human airway smooth muscle.

**Figure 6.**
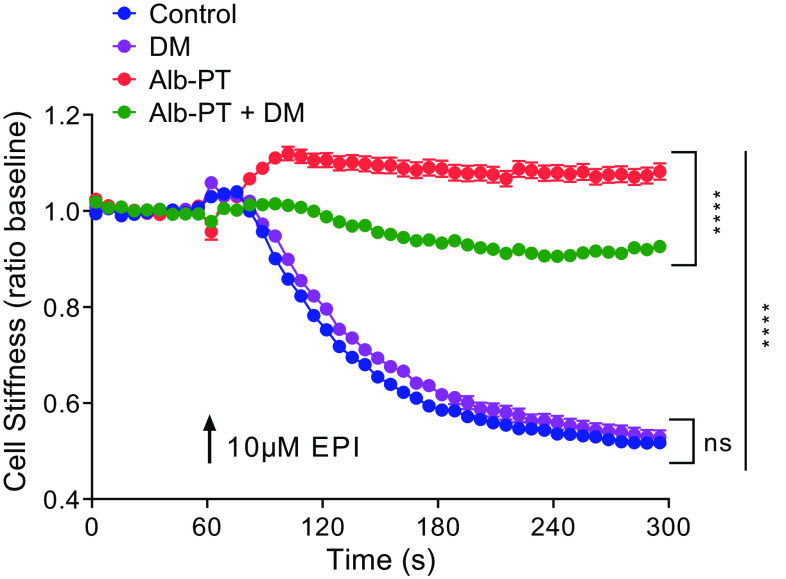
EPI induces HASM cell shortening under experimental conditions that induce β_2_AR desensitization. HASM cells were treated for 18 h with or without 10 µM albuterol. After 18 h, cells were washed 3× with fresh media, incubated for 20 min with magnetic beads, and washed again 3× with fresh media to remove unbound beads, before stimulation with 10 µM EPI. For each individual cell, baseline stiffness was measured for first 60 s, and changes in the stiffness in response to EPI were measured continuously for the next 240 s (EPI was added at 60 s). For α_1_AR blockade, Alb-PT and diluent treated cells were treated for 10 min with 1 µM DM, followed by stimulation with 10 µM EPI. Data are presented as means ± SE (*n* = 295–389 individual cells per condition). Analyses were done by using one-way ANOVA, followed by Tukey’s multiple comparison tests. ****P = 0.0001. α_1_AR, α_1_-adrenergic receptor; β_2_AR, β_2_-adrenergic receptor; DM, doxazosin mesylate; EPI, epinephrine; HASM, human airway smooth muscle.

To ascertain whether EPI can act as a procontractile agonist via activating α_1_ARs expressed on HASM cells, we assessed single-cell calcium and single-cell shortening methods as surrogates for HASM contraction (37). Under experimental conditions that induce β_2_AR desensitization (as above), we found that pharmacological inhibition of α_1_ARs with DM significantly attenuated EPI-induced intracellular calcium mobilization (Fig. 5); DM had little effect on EPI-induced [Ca^2+^]_i_ in nondesensitized HASM cells. To address the physiological consequences of α_1_AR activation with EPI, magnetic twisting cytometry (MTC) was applied to quantify changes in the cytoskeletal stiffness of HASM cells (34, 36). In nondesensitized HASM cells, EPI decreased the cell stiffness (i.e., HASM cell relaxation); and, EPI-induced relaxation was not affected by DM pre-treatment (Fig. 6). In contrast, upon β_2_AR desensitization, EPI increased the cell stiffness (i.e., HASM cell contraction), which was abrogated by pretreating the cells with DM (Fig. 6). These results suggest that, under specific conditions of β_2_AR tachyphylaxis in asthma, endogenous or exogenous EPI can preferentially activate α_1_ARs and evoke intracellular calcium, myosin light chain phosphorylation, and HASM cell shortening. These data also suggest a feed-forward mechanism via two distinct classes of GPCRs to reinforce bronchoconstriction (e.g., exercise-induced or emotional stress) in patients with β_2_-agonist insensitivity.

### Epinephrine Induces Bronchoconstriction after Desensitization of the β2AR

**Figure 7.**
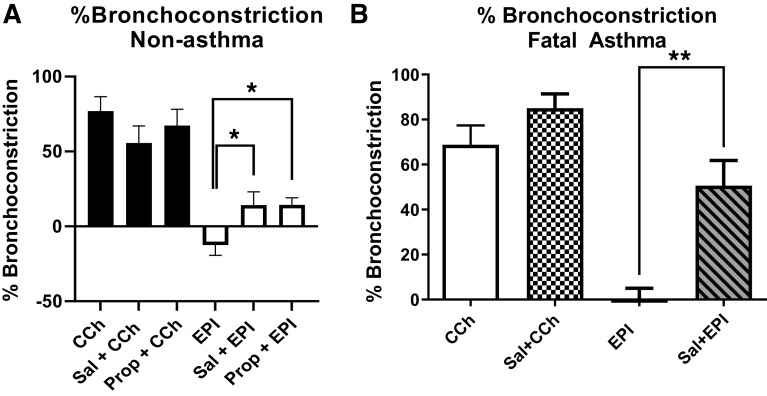
Epinephrine induces bronchoconstriction after β_2_AR tachyphylaxis. EPI (10 μM, 10 min) induced bronchodilation at baseline and EPI-induced bronchoconstriction after salmeterol-induced β_2_AR tachyphylaxis (1 μM, 48 h pretreatment) or β_2_AR blockade with propranolol (10 μM, 30 min pretreatment) in both nonasthma hPCLS (*A*) and fatal asthma hPCLS (*B*). Carbachol (CCh)-induced bronchoconstriction (10 μM, 10 min) used as a positive control to confirm contractility of hPCLS (values are means ± SEM, *n* = 8 slices from one representative nonasthma donor, *n* = 5 slices from one representative fatal asthma donor) (*P ≤ 0.05, **P ≤ 0.01). β_2_AR, β_2_-adrenergic receptor; DM, doxazosin mesylate; EPI, epinephrine; HASM, human airway smooth muscle; hPCLS, human precision cut lung slices; Prop, propranolol; Sal, salmeterol.

To examine whether EPI induced bronchoconstriction in an intact airway, human small airways in hPCLS from one nonasthma and one fatal asthma donor were treated with EPI before and after β_2_AR desensitization. EPI induced bronchodilation at baseline; however, after salmeterol-induced β_2_AR desensitization or β_2_AR blockade with propranolol, EPI induced bronchoconstriction (Fig. 7*A*). EPI induced greater bronchoconstriction in the fatal asthma donor-derived hPCLS as seen in Fig. 7*B*. These data suggest that EPI works as a bronchodilator at baseline, but switches to a bronchoconstrictor after β_2_AR tachyphylaxis or blockade.

## DISCUSSION

**Figure 8.**
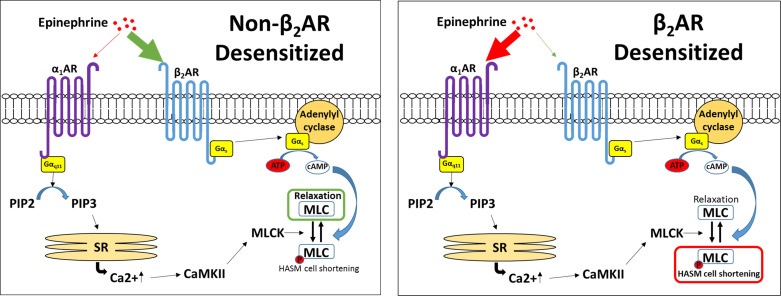
Alterations in β_2_AR and α_1_AR signaling pathways. Under normal, nondesensitized conditions, EPI preferentially binds the β_2_AR, leading to increases in cAMP and dephosphorylation of MLC to induce relaxation. Following desensitization of the β_2_AR, EPI preferentially binds the α_1_AR, inducing increased cytosolic calcium flux and pMLC, inducing bronchoconstriction. α_1_AR, α_1_-adrenergic receptor; β_2_AR, β_2_-adrenergic receptor; cAMP, 3′,5′-cyclic adenosine monophosphate; EPI, epinephrine; pMLC, myosin light chain phosphorylation.

Here we demonstrated that, under experimental conditions that induce β_2_AR tachyphylaxis, EPI evoked calcium mobilization, myosin light chain phosphorylation, and bronchoconstriction by activating α_1_ARs expressed on HASM cells (Fig. 8). Immunohistochemistry studies showed that α_1_ARs are expressed in the smooth muscle layers of intact human airways. Of note, in isolated primary HASM cells, we detected a high abundance of *ADRA1B* transcripts (the α_1_AR subtype B)—but not *ADRA1A* (the α_1_AR subtype A)—which were approximately 10× higher than that of *ADRB2* (encoding β_2_AR). These results are consistent with selective β_2_AR agonism of EPI (i.e., generated endogenously or administered parenterally) and support the concept of spare α_1_-adrenoceptors on HASM cells (41, 42). Our findings also address a long-standing question of α_1_AR expression and function in HASM cells (22–24) and provide a basis for reexamining α_1_AR inhibition for the management of stress/exercise-induced asthma and/or β_2_-agonist insensitivity in patients with difficult-to-control, disease subtypes.

Epinephrine (or adrenaline), an endogenous catecholamine involved in the body’s fight-or-flight responses to stress, activates α_1_ARs expressed on various organs to evoke a wide range of physiological functions, including vasoconstriction (8, 9). In the smooth muscle of human bronchi, however, the physiological effect of EPI on α_1_ARs remains unclear. Evidence suggests that EPI promotes bronchodilation by stimulating β_2_ARs expressed on HASM (12, 39). As shown in Figs. 3, 4, 5, and 6, under normal, non-β_2_AR-desensitized conditions, EPI induced HASM cell relaxation, which was not affected by an α_1_AR antagonist, doxazosin mesylate. In contrast, upon β_2_AR desensitization, EPI evoked HASM cell contraction in an α_1_AR-dependent manner (Figs. 3, 4, 5, and 6). In intact human small airways, EPI also evoked airway constriction in the face of β_2_ desensitization or blockade (Fig. 7). This “switch-like” activation of α_1_ARs, and related EPI-induced biochemical and mechanical signals, also emerged upon pharmacological inhibition of β_2_ARs (Fig. 2). These results support previous studies examining the effects of α_1_AR activation on smooth muscle contraction (20, 21). Furthermore, our findings are consistent with the findings of the study by Naline et al. (43), showing decreased adrenaline-induced relaxation of human bronchi after 1 h salmeterol pretreatment. Taken together, these studies now identify specific conditions, in which an endogenous catecholamine (EPI) can induce a “switch-like” activation of α_1_ARs, suggesting a previously unrecognized feed-forward mechanism driving bronchospasm via two distinct classes of G protein-coupled receptors (GPCRs).

Interestingly, HASM cells were enriched with transcript levels of *ADRA1B* (the α_1_AR subtype B), but not *ADRA1A* (the α_1_AR subtype A) (Fig. 1*A*). Since α_1_AR subtype-specific antibodies are unavailable (44), whether α_1_-adrenoceptor subtype protein expression exists on HASM cells remains unknown. We, however, chose a commercially available antibody against α_1_ARs and performed immunohistochemistry studies. Staining of the α_1_AR was readily visualized in both intact airways and isolated HASM cells (Fig. 1*B*). Using intact airways, we detected appreciable α_1_AR expression in both epithelial and smooth muscle layers that was unaltered in asthma. Our future studies will investigate the distribution of α_1_AR subtypes and, using siRNA-mediated knockdown and/or subtype-specific inhibition approaches, determine the specificity and selectivity of receptor subtype that is activated by EPI in HASM cells. Our data collectively showed the presence of α_1_ARs on HASM cells and EPI-induced mechanical reinforcement of HASM shortening is α_1_AR-dependent.

Previous studies have implicated the α_1_AR in exercise-induced asthma (22, 23, 45), as exercise increases the release of adrenalines (epinephrine and norepinephrine) that, like the fight-or-flight responses to stress, evoke a wide range of physiological responses, including vasoconstriction. Of note, an asthma exacerbation is a high-stress event. Accordingly, overflow of EPI and NE in circulation could stimulate airway smooth muscle given the spatial proximity of the airways to the vasculature, including the nerves that innervate them (46). EPI levels can also be elevated in individuals who receive EPI injections as part of the treatment for anaphylaxis. Normally, circulating EPI would induce bronchodilation via the β_2_AR; however, if an individual has been repeatedly using β_2_-agonists (short- and long-acting combined) for relief of bronchospasm, tachyphylaxis of the β_2_AR can occur, increasing the probability of EPI activating the α_1_AR system and evoking bronchoconstriction.

Our study suggests that stress/exercise-induced release of EPI may evoke bronchoconstriction in patients with difficult-to-control or β_2_-agonist insensitivity. Of note, Inman and O’Byrne (47) in their study found that four times daily use of albuterol for 1 wk worsened exercise-induced bronchoconstriction compared with placebo, which may, in part, be due to endogenous EPI release and activation of the α_1_AR due to repeated albuterol-induced desensitization of the β_2_AR. We posit that mortalities resulting from this may be underreported due to EPI being reported as ineffective rather than potentially worsening asthma. As such, further studies are warranted to investigate the therapeutic value of α_1_AR blockade, specifically in individuals exhibiting airflow obstruction due to stress/exercise and/or therapy-resistance to β_2_-agonists.

Interestingly, glucocorticoids increase α_1b_AR mRNA (48), suggesting that inhaled steroids may contribute to altering the balance between β_2_AR- and α_1_AR-dependent signaling in airway smooth muscle in response to EPI. In addition, activation of α_1_AR and/or α_2_AR has been shown to induce the proliferation of airway smooth muscle in rabbits that was inhibited by adenylate cyclase activation (49). Taken together, these studies suggest that multiple mechanisms by which α_1_AR may play a role in asthma.

In conclusion, we demonstrated that blockade of the α_1_AR abrogated EPI-induced calcium flux, pMLC, and HASM shortening, under experimental conditions that induce β_2_AR desensitization, providing a basis for reexamining α_1_AR inhibition for the management of stress/exercise-induced asthma and/or β_2_-agonist insensitivity in patients with difficult-to-control, disease subtypes.

## DATA AVAILABILITY

Data will be made available upon reasonable request.

## SUPPLEMENTAL DATA

10.6084/m9.figshare.19617252 Supplemental Table S1 and Supplemental Fig. S1: https://doi.org/10.6084/m9.figshare.19617252.

## GRANTS

This work was supported by the New Jersey Alliance for Clinical and Translational Science (UL1TR0030117) and the National Institutes of Health grants (P01HL114471 and R56HL155937).

## DISCLOSURES 

No conflicts of interest, financial or otherwise, are declared by the authors.

## AUTHOR CONTRIBUTIONS

B.T.D., G.C., S.S.A., and R.A.P. conceived and designed research; B.T.D., G.C., S.O., J.L., M.K., B.E.H., and V.P. performed experiments; B.T.D., G.C., S.O., J.L., M.K., B.E.H., C.J.K.-W., S.S.A., and R.A.P. analyzed data; B.T.D., G.C., S.O., J.L., V.P., C.J.K.-W., S.S.A., and R.A.P. interpreted results of experiments; B.T.D., G.C., J.L., S.S.A., and R.A.P. prepared figures; B.T.D., G.C., C.J.K.-W., S.S.A., and R.A.P. drafted manuscript; B.T.D., G.C., S.O., J.L., M.K., B.E.H., V.P., C.J.K.-W., S.S.A., and R.A.P. edited and revised manuscript; B.T.D., G.C., S.O., J.L., M.K., B.E.H., V.P., C.J.K.-W., S.S.A., and R.A.P. approved final version of manuscript.
